# Defining responders to therapies by a statistical modeling approach applied to randomized clinical trial data

**DOI:** 10.1186/s12916-019-1345-2

**Published:** 2019-06-18

**Authors:** Francesca Bovis, Luca Carmisciano, Alessio Signori, Matteo Pardini, Joshua R. Steinerman, Thomas Li, Aaron P. Tansy, Maria Pia Sormani

**Affiliations:** 1 Department of Health Sciences (DISSAL), University of Genova, Via Pastore 1, 16132 Genova, Italy; 2 Department of Neuroscience, Rehabilitation, Ophthalmology, Genetics, Maternal and Child Health, University of Genoa, Genoa, Italy; 3 IRCCS Ospedale Policlinico San Martino, Genova, Italy; 4 Teva Pharmaceuticals, Malvern, PA USA

**Keywords:** Multiple sclerosis, Personalized treatment effects, Personalized medicine, Clinical trials

## Abstract

**Background:**

Personalized medicine is the tailoring of treatment to the individual characteristics of patients. Once a treatment has been tested in a clinical trial and its effect overall quantified, it would be of great value to be able to use the baseline patients’ characteristics to identify patients with larger/lower benefits from treatment, for a more personalized approach to therapy.

**Methods:**

We show here a previously published statistical method, aimed at identifying patients’ profiles associated to larger treatment benefits applied to three identical randomized clinical trials in multiple sclerosis, testing laquinimod vs placebo (ALLEGRO, BRAVO, and CONCERTO). We identified on the ALLEGRO patients’ specific linear combinations of baseline variables, predicting heterogeneous response to treatment on disability progression. We choose the best score on the BRAVO, based on its ability to identify responders to treatment in this dataset. We finally got an external validation on the CONCERTO, testing on this new dataset the performance of the score in defining responders and non-responders.

**Results:**

The best response score defined on the ALLEGRO and the BRAVO was a linear combination of age, sex, previous relapses, brain volume, and MRI lesion activity. Splitting patients into responders and non-responders according to the score distribution, in the ALLEGRO, the hazard ratio (HR) for disability progression of laquinimod vs placebo was 0.38 for responders, HR = 1.31 for non-responders (interaction *p* = 0.0007). In the BRAVO, we had similar results: HR = 0.40 for responders and HR = 1.24 for non-responders (interaction *p* = 0.006). These findings were successfully replicated in the CONCERTO study, with HR = 0.44 for responders and HR=1.08 for non-responders (interaction *p* = 0.033).

**Conclusions:**

This study demonstrates the possibility to refine and personalize the treatment effect estimated in randomized studies by using the baseline demographic and clinical characteristics of the included patients. The method can be applied to any randomized trial in any medical condition to create a treatment-specific score associated to different levels of response to the treatment tested in the trial. This is an easy and affordable method toward therapy personalization, indicating patient profiles related to a larger benefit from a specific drug, which may have implications for taking clinical decisions in everyday clinical practice.

## Introduction

The results of clinical trials comparing a new treatment with a control via a randomized procedure are based on an overall summary measure over the whole population enrolled. Assessing the individual patient response to a new treatment is not possible, since each study subject is assigned to receive either the new treatment or the control, but not both. That is, the treatment effect is not observable at an individual level. The treatment efficacy is expressed by a single number (the treatment effect size) that is assumed to apply to each treated patient, but this treatment effect can vary according to specific characteristics of the patients enrolled. The aim of personalized medicine is the tailoring of medical treatment to the individual characteristics of each patient in order to optimize individuals’ outcomes. The key issue for personalized medicine is finding the criteria for an early identification of patients who can be responders and non-responders to each therapy. In this study, we present a powerful statistical modeling approach previously developed by Zhao et al. [1], aimed at identifying subgroups of patients with the largest benefit from a therapy tested in a randomized clinical trial, by a multivariate post hoc analysis of baseline patients’ characteristics.

The standard analytical approach to characterize patients with the largest benefits from a treatment is based on post hoc subgroup analyses, which tests the treatment effect on one or more dichotomized baseline variables. This procedure, however, may not be efficient, especially when the number of baseline variables is large. Further, findings from post hoc subgroup analyses need to be validated in independent studies.

Various novel quantitative methods have been proposed to deal with the identification of factors associated to heterogeneous treatment effects, both in cases of single covariates [2–5] and in those of multiple covariates [6]. In 2013, Zhao et al. [1] proposed a simple modeling approach to build a continuous score made up of multiple baseline covariates from a randomized clinical trial to efficiently identify patients with different levels of treatment benefit. This approach has some advantages: it is very simple from the computational point of view since it is based on the difference between two standard multivariate prognostic models (on the control and on the experimental arm); it generates a linear score made of baseline variables, which can be calculated in a post hoc analysis with no additional costs; when large clinical trials, or, even better, multiple trials testing the same drug are available, it is possible to replicate the results to validate them. The method’s original application was to improve enrichment strategies for future randomized controlled trials. The aim of this paper is to present the application of such a methodology to build a treatment-specific algorithm for potential use in making treatment decisions in clinical practice.

We used here, as a working example, three large clinical trials in multiple sclerosis (MS) who failed to bring the tested drug to approval. This is an ideal scenario, since having three identical trials allows for a rigorous testing–validation–external validation procedure. The approach proposed here can be easily extended to the post hoc analysis of any trial in any medical field. This methodology, without additional cost beyond that of the trials themselves, will indicate the characteristics of individual subjects with higher/lower benefits from any specific treatment, providing patients and clinicians with insights into personalized treatment options.

## Methods

### Background

We used for the present analysis three large randomized clinical trials in relapsing–remitting (RR) MS that tested the efficacy of laquinimod vs placebo. Laquinimod is an orally available carboxamide derivative developed for RRMS. Its mechanism of action may comprise immunomodulatory effects on T cells, monocytes, and dendritic cells as well as neuroprotective effects with prominent actions on astrocytes. Laquinimod was tested in phase II and III clinical trials in RRMS at different dosages initially ranging from 0.1 to 0.6 mg/day. The compound was well tolerated, yet the dosages tested only led to moderate effects on the reduction of relapse rates as a primary study endpoint in two phase III trials (ALLEGRO [7] and BRAVO [8]). In contrast, significant effects on brain volume and disease progression were observed. The Committee for Medicinal Products for Human Use (CHMP) refused marketing authorization for RRMS based on the assessment of the risk–benefit ratio with regard to data from mechanistic and animal studies. A third clinical trial (CONCERTO [9]) was run with disability progression as the primary endpoint. The trial was negative on this endpoint, and the laquinimod program for RRMS was not continued. The analysis presented here is therefore focused on showing the power of the methodology to identify responders to a therapy, taking advantage from the ideal setting of three large clinical trials, and showing that even a drug whose efficacy was not confirmed on the overall population can give large benefits on a specific subgroup of patients.

### Patients and study design

This is a post hoc analysis of three randomized clinical trials, the ALLEGRO, BRAVO, and CONCERTO studies (ClinicalTrials.gov identifiers: NCT00509145, NCT00605215, and NCT01707992, respectively). The study design and inclusion/exclusion criteria of the trials were the same for the three studies and have been described elsewhere [7–9]. Briefly, eligibility criteria included age 18–55 years, diagnosis of RRMS (revised McDonald criteria [10]), Expanded Disability Status Scale (EDSS) scores of 0–5.5, and presence of relapse activity in the previous 12–24 months.

### Primary endpoint

The primary endpoint of this analysis is the disability progression as defined in the three trials. A progression event was defined as 1.0 point increase in the EDSS score if baseline score was between 0 and 5.0, or a 0.5 point increase if baseline score was 5.5, sustained for 3 months.

### Statistical analysis

According to the accepted methodology of building and validating a prediction rule, a training set (model creation), a validation set (model performance evaluation and refinement toward finalization), and an external validation set not utilized in the model creation (final model validation) are needed [11].

Therefore, in this study, the model predicting response to therapy was developed in these three steps:

1. A set of scores was created on the training set (trial 1 = ALLEGRO)
2. The best score was chosen according to the performance on the validation set (trial 2 = BRAVO)
3. The score was externally validated on the third independent validation set (trial 3 = CONCERTO)

#### Building the score on the training set

The score building followed the modeling approach described by Zhao et al. [1].

For each subject *i*, we can observe the four values (T*i*, Y*i*, A*i*, Z*i*), where T*i* is the time to disability progression, Y*i* is the progression event (yes/no), A*i* is the treatment arm (here a binary variable: PL = placebo and AT = active treatment), and Z*i* is the covariate vector (Z_1i_, Z_2i_,...,Z_pi_) made of the values of all the baseline characteristics. By fitting two separate Cox models one for each arm, we obtain the following models:

$$ \log \left({h}_{\mathrm{PL}}(t)\right)=\log \left({h}_{0\mathrm{PL}}(t)\right)+\left(\ {\beta}_{1\mathrm{PL}}\bullet {Z}_1+{\beta}_{2\mathrm{PL}}\bullet {Z}_2+\cdots +{\beta}_{p\mathrm{PL}}\bullet {Z}_p\right) $$

$$ \log \left({h}_{\mathrm{AT}}(t)\right)=\log \left({h}_{0\mathrm{AT}}(t)\right)+\left({\beta}_{1\mathrm{AT}}\bullet {Z}_1+{\beta}_{2\mathrm{AT}}\bullet {Z}_2+\cdots +{\beta}_{p\mathrm{AT}}\bullet {Z}_p\right) $$

where *h*(*t*) is the hazard function.

Assuming a common baseline hazard function *h*_0_(*t*) = *h*_0AT_(*t*) = *h*_0PL_(*t*), due to the randomized nature of the two groups, it is possible to calculate a difference score:

$$ D(Z)=\log (HR)=\left({\beta}_{1\mathrm{AT}}-{\beta}_{1\mathrm{PL}}\right)\bullet {Z}_1+\left({\beta}_{2\mathrm{AT}}-{\beta}_{2\mathrm{PL}}\right)\bullet {Z}_2+\cdots +\left({\beta}_{p\mathrm{AT}}-{\beta}_{p\mathrm{PL}}\right)\bullet {Z}_p $$

The difference score *D*(*Z*) is the patient-specific score (*Z* representing the set of patient-specific covariates), predicting the size of treatment effect according to his/her profile.

We selected the baseline variables collected in all the three trials to build the response score, as:

- Age (continuous OR in 3 groups with cut-offs: < 20, 20–50, 50+)
- Disease duration (continuous, log transformed)
- Sex (binary)
- EDSS (continuous OR binary with cut-off 4)
- Relapses in the previous year (continuous)
- T2 lesion volume (continuous, log transformed)
- T1 lesion volume (continuous, log transformed)
- Normalized brain volume (NBV) (continuous)
- Presence of gadolinium-enhancing (Gd+) lesions (binary)

The model building described so far was detailed in the Zhao et al. [1] paper. We expanded the proposed methodology adding a strategy for the best model selection, based on an unsupervised approach. We generated all the possible combinations of the 9 baseline variables, building 511 models (2^9^ − 1) on the placebo and on the treated group, according to the procedure described above. This number was multiplied by 4 considering the different coding of age and EDSS, for a total number of 2044 response scores.

Then, we ranked these score_*j*_ (*j* = 1,…,2044) for their ability to identify patients with heterogeneous response to treatment by ranking the *p* values of the treatment by score_*i*_ interaction in Cox models including treatment, score_*j*_, and treatment by score_*j*_ interaction. We selected all the scores giving a *p* value for interaction with treatment < 0.05; this cut-off was arbitrarily defined to screen the scores. This procedure indicated a first set of candidate response models on the training set.

#### Analysis on the validation set

The *n* models with a *p* value for interaction < 0.05 on the training set were selected and tested on the validation set. The models were ranked in ascending order according to the *p* values for treatment by score interaction on the validation set. The first five models (that is, the model with rank = 1 to 5) on the validation set were selected as the candidate models to predict response to therapy. To choose one model among these five scores, we merged the training and the validation set, re-estimating the five models’ coefficients. The score with the lower *p* value for treatment by score interaction on the merged dataset was chosen as the final model. This procedure is based on the performances on the validation set of the models defined on the training set; the final model will be the one among the best 5 on the validation set with the best performance on the integrated training–validation set. To evaluate the consistency of the model choice, the area under the AD(*q*) curve [1] was calculated.

Zhao et al. [1] defined the AD(*c*) curve as the curve reporting the average treatment effect (that is the laquinimod vs placebo hazard ratio (HR)) in the subgroup of patients with difference score *D*(*Z*) ≤ *c*, where *c* represents the response score values ranked in ascending order [1]. In other words, the AD(*c*) curve reports the treatment effect in subgroups of patients with score ≤ *c*, for each *c*. To compare two different scoring systems *D*_1_ and *D*_2_, the AD(*c*) curve must be transformed to the AD(*q*) curve, where *q* represents the proportion of patients with score ≤ *c* [1]. The area under the AD(*q*) curve would be equal to the overall HR in case of a perfectly uniform treatment effect in every subgroup of patients (defined as the *q*% of the overall population). The lower the area under the AD(*q*) curve, the higher the heterogeneity of treatment effect according to different levels of the score. Technical details about the definition and the properties of the AD(*q*) are reported in the cited paper [1].

#### Cut-off choice

Once the best model was selected, we choose a cut-off to visualize the treatment effect in patients defined as responders and non-responders. The cut-off was chosen by a visual inspection of the AD(*q*) curve of the selected model, as the point of change of slope. Also, the shape of the distribution of the score was evaluated. This distribution, in fact, represents the predicted distribution of responses to the drug according to the baseline profile in all the patients. The shape and the width of the distribution can give indication about the heterogeneity of response to the assessed drug and can help identify a possible cut-off point separating responders vs non-responders. Once a range of candidate cut-offs was identified by a visual inspection of the AD(*q*) curve and of the score distribution, a systematic procedure testing all the possible cut-off values in the identified range of values was run, choosing the one dividing the cohort in two subgroups with the lowest *p* value for treatment by subgroup interaction on the training set.

#### Final external validation

The score calculated on the training/validation set was then tested on the external dataset. The *p* value for the score by treatment interaction (indicating whether the treatment has a different efficacy according to different values of the score) was calculated. The dichotomized score (according to the previously defined cut-off) was tested, and HR and 95% confidence intervals (95% CI) in the non-responders and in the responders group were evaluated and compared. A final evaluation of the differential treatment effect in responders vs non-responders on all the three datasets was carried out by a test for homogeneity of treatment effects in each subgroup across the three studies.

## Results

### Summary of overall clinical trial results

Two arms were included from all studies: placebo and laquinimod 0.6 mg daily. Complete clinical and demographic baseline data were available for 1101/1106 (99.6%) patients from ALLEGRO study, 881/884 (99.7%) from BRAVO study, and 1456/1467 (99.3%) from CONCERTO study, for a total sample of 3438 patients. Table 1 reports the baseline characteristics of the patients included in this analysis.

**Table 1:** Baseline demographic and clinical characteristics of the population enrolled in each of the three clinical trials included in the analysis

	ALLEGRO *N* = 1101	BRAVO *N* = 881	CONCERTO *N* = 1456	*p*
Age (SD), years	38.69 (9.14)	37.31 (9.39)	36.37 (9.10)	< 0.001
Gender, number of males (%)	345 (31.3)	280 (31.8)	465 (31.9)	0.947
Mean number of relapses in the previous year (SD)	1.25 (0.69)	1.30 (0.63)	1.32 (0.55)	0.018
Mean disease duration (SD), years	8.63 (6.79)	6.80 (6.29)	5.81 (4.07)	< 0.001
Median EDSS (range)	2.5 (0.0–6.0)	2.5 (0.0–5.5)	2.5 (0.0–5.5)	0.281
Number of patients with baseline Gd+ scan (%)	475 (43.1)	320 (36.3)	578 (39.7)	0.008
Mean T2 lesion volume (SD) (log-transformed cm^3^)	1.70 (1.26)	1.47 (1.42)	1.60 (1.22)	< 0.001
Mean T1 lesion volume (SD) (log-transformed cm^3)^	−0.09 (1.93)	0.02 (1.87)	1.21 (1.31)	< 0.001
Mean normalized brain volume (SD), cm^3^	1581.8 (93.2)	1584.0 (94.4)	1435.0 (93.0)	< 0.001

Data reported as mean (standard deviation) and number (percentage) for continuous and categorical variables, respectively (unless otherwise specified)

*EDSS* Expanded Disability Status Scale, *GD+* gadolinium-enhancing

Overall, laquinimod reduced the disability progression as compared to placebo by 36% (HR = 0.64, 95% CI 0.45–0.91, *p* = 0.01) in the ALLEGRO study, by 31% (HR = 0.69, 95% CI 0.46–1.02, *p* = 0.06) in the BRAVO study, and by 6% in the CONCERTO study (HR = 0.94, 95% CI 0.65–1.35, *p* = 0.72).

### Score building on the training set and score selection on the validation set

**Fig. 1:**
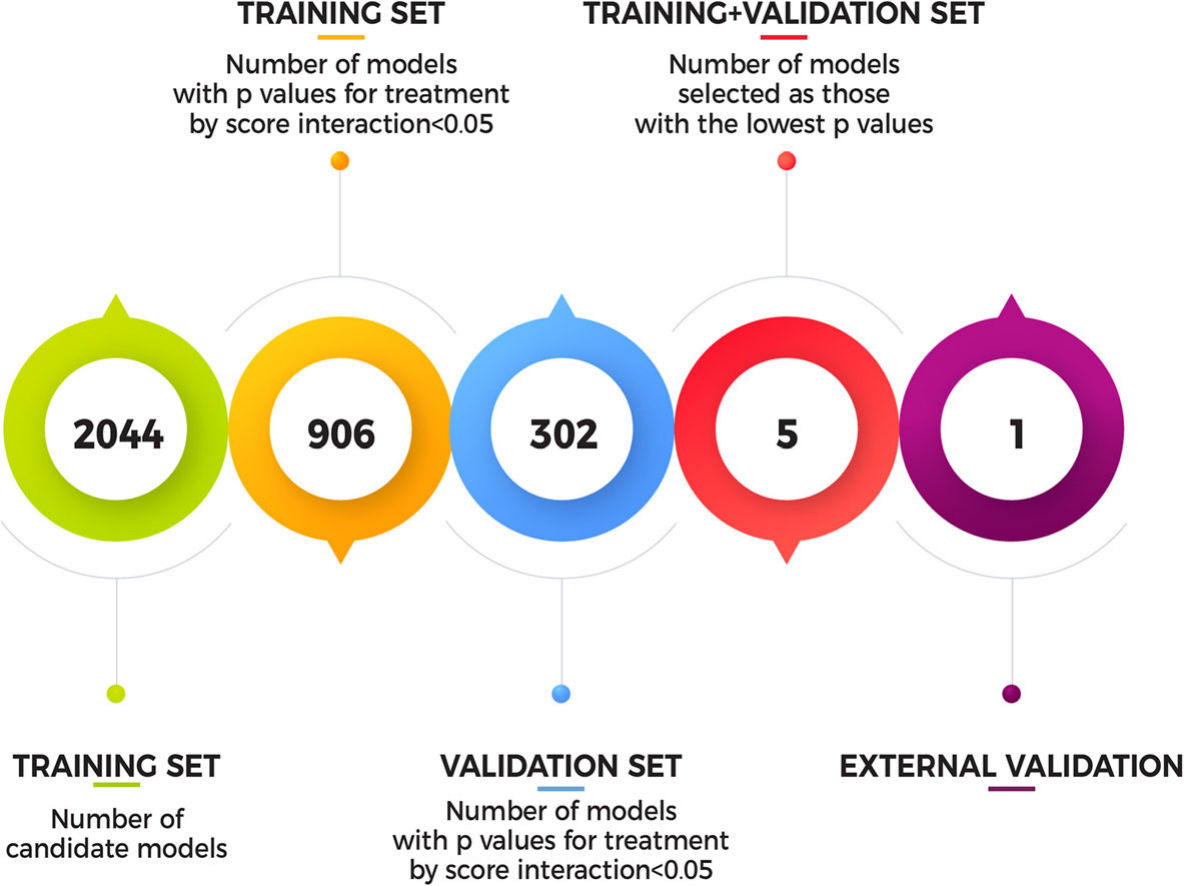
Flow chart of the process to define the response score using the training (ALLEGRO trial), the validation (BRAVO trial), and the external validation (CONCERTO trial) approach

The full procedure for building and validating the response score is described in Fig. 1.

All 2044 response scores from all possible combinations of the selected baseline variables were generated and tested on the training set (ALLEGRO trial). The scores were generated according to the methodology aforementioned in the “Methods” section with respect to their ability to discriminate different levels of response to treatment. Of the 2044 models generated, 906 had a *p* value for score by treatment interaction < 0.05 on the training set. Of these, 302 had a *p* value for treatment interaction on the validation set < 0.05 (BRAVO). The first five models ranked according to the *p* for interaction on the validation set are reported in Table 2 (the list of all the 2044 response scores are available upon request).

**Table 2:** List of the five best models obtained defining the models on the training set and testing them on the validation set, ordered according to the *p* value for treatment by score interaction on the merged training and validation datasets

Model	p* training set	p* validation set	p* training + validation set	Area under the AD(*q*) curve
Response score 1 = − 0.38 × Age3 + 0.65 × sex + 0.39 × Rel − 0.002 × NBV − 0.20 × Gd+	0.026	0.004	0.00027	0.358
Response score 2 = 0.61 × sex + 0.37 × Rel − 0.002 × NBV − 0.23 × Gd+ − 0.02 × age + 0.02 × EDSS	0.037	0.003	0.00031	0.368
Response score 3 = 0.05 × EDSS4 + 0.61 × sex + 0.37 × Rel − 0.002 × NBV − 0.22 × Gd+ − 0.02 × age	0.040	0.004	0.00032	0.368
Response score 4 = 0.61 × sex + 0.37 × Rel − 0.002 × NBV − 0.22 × Gd+ − 0.02 × age	0.042	0.003	0.00033	0.369
Response score 5 = 0.63 × sex + 0.36 × Rel − 0.01 × age + 0.02 × EDSS	0.037	0.004	0.00044	0.356

Area under the AD(*q*) curve represents the curve generated by plotting the cumulative distribution of patients ranked by individual treatment response score and the overall treatment effect relative to a given proportion of patients. The lower is the curve, the higher the heterogeneity of treatment effect. Age3: Age3 = 1 if age < 20 years, Age3 = 2 if age in the range 20–50, Age3 = 3 if age ≥ 50 years; sex = 1 if sex = male and sex = 0 if sex = female, EDSS4 = 0 if EDSS  4, EDSS4 = 1 if EDSS ≥ 4

*Rel* number of relapses in the previous year; *NBV* normalized brain volume, in cubic centimeter; *Gd+* gadolinium-enhancing; *EDSS* Expanded Disability Status Scale; *age* age in years

*p for treatment by score interaction

As the five models reflect equivalent discriminant ability on the validation set, these were re-fitted on the merged training–validation set. The five resulting scores were then ranked, and the one with the lowest *p* for interaction (*p* = 0.00027) with treatment was chosen.

The final selected response score was the following:

$$ \mathrm{Response}\ \mathrm{score}=-0.38\times \mathrm{age}\ \left(\mathrm{age}<20\ \mathrm{years}=1,\mathrm{age}\ \mathrm{between}\ 20\ \mathrm{and}\ 50=2,\mathrm{age}>50\ \mathrm{years}=3\right)+0.65\times \mathrm{sex}\ \left(\mathrm{male}=1,\mathrm{female}=0\right)+0.39\times \mathrm{relapses}\ \mathrm{in}\ \mathrm{the}\ \mathrm{previous}\ \mathrm{year}\ \left(\mathrm{number}\right)-0.002\times \mathrm{normalized}\ \mathrm{brain}\ \mathrm{volume}\ \left({\mathrm{cm}}^3\right)-0.20\times \mathrm{presence}\ \mathrm{of}\ \mathrm{Gd}+\mathrm{activity}\ \left(\mathrm{yes}=1,\mathrm{no}=0\right) $$

**Fig. 2:**
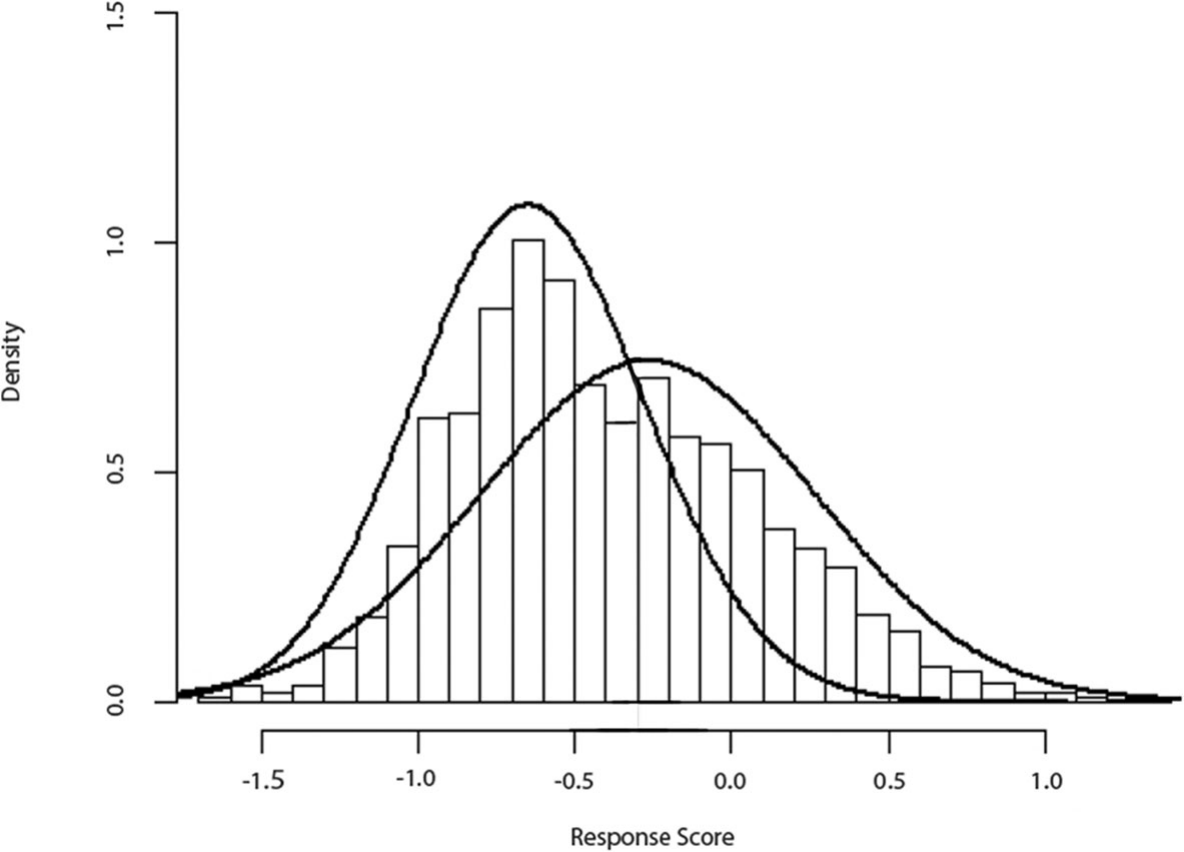
Histogram of the response score distribution, representing the distribution of the predicted response to the drug, with the two normal curves giving the best fit to the score distribution

A constant value was added to the score (*k* = 3.44), in order to obtain a mean response score equal to the overall logHR. The score can be then interpreted as the response to the drug predicted by the model for each patient. The distribution of the response score is reported in Fig. 2.

**Fig. 3:**
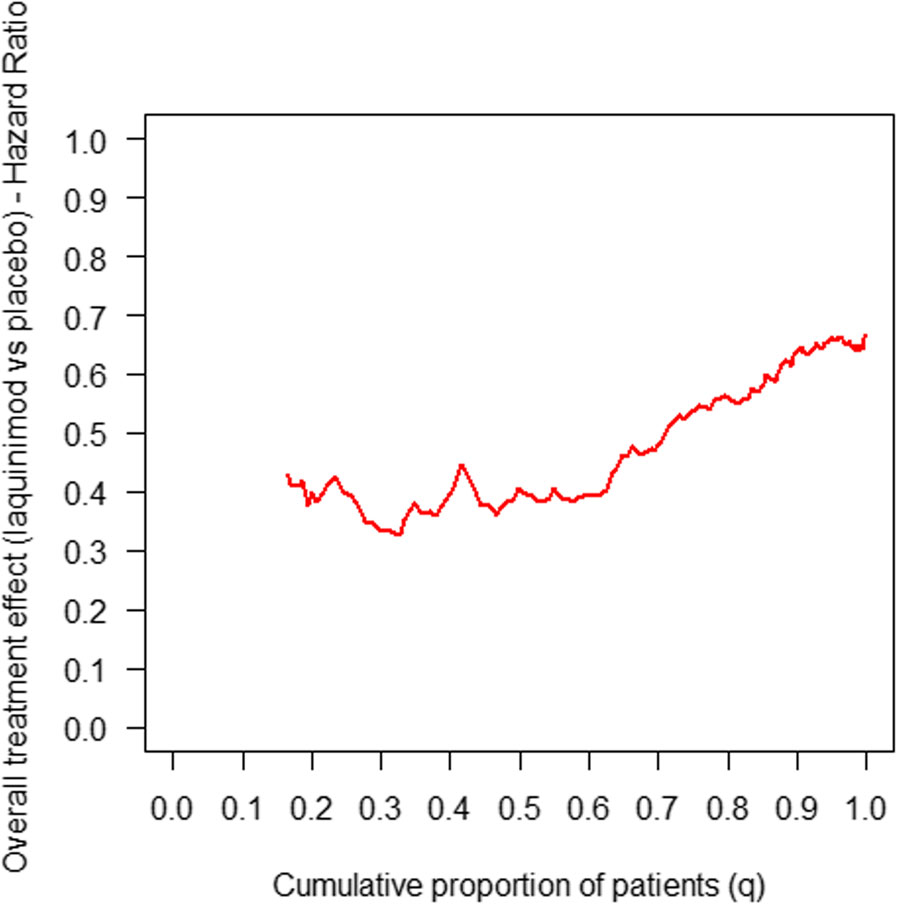
Treatment effect (hazard ratio (HR), on the *y* axis) in the cumulative percentage of patients ranked by increasing values of the response score (*x* axis). The HR is estimated on the increasing *q*% of patients ranked by increasing values of the response score. Therefore, the HR for a *q* = 0.5 represents the HR in the 50% of subjects with the lower score, while the HR for a *q* = 1 represents the HR of the whole cohort (100% of the subject enrolled)

The distribution is not normal but is skewed to the right and is better fitted by a mixture of two normal distributions. The shape of the response score distribution suggests the presence of responders and non-responders to the drug. This was confirmed by the AD(*q*) curve (Fig. 3), showing a change of curve slope.

The best score resulted to be:

$$ \mathrm{cut}-\mathrm{off}=-0.31 $$

We then tested the score with this definition of responders (those patients with a response score ≤ − 0.31) and non-responders (those patients with a response score > − 0.31) on the trial’s dataset. The proportion of responders was 61.3% (*N* = 675) in the ALLEGRO and 58.3% (*N* = 514) in the BRAVO datasets. The treatment effect in the subgroups defined as responders and non-responders are reported in Table 3.

**Table 3:** Response to treatment by treatment response score subgroups in the three clinical trials

Trial	Score group	Number of patients (%)			Cumulative probability of 2-year EDSS progression		Treatment effect	
		Total	Placebo	Laquinimod	Placebo	Laquinimod	HR (95% CI)	p*
ALLEGRO *N* = 1101	NR	426 (38.7)	219 (51.4)	207 (48.6)	6.30	8.87	1.31 (0.77–2.25)	0.0007
	R	675 (61.3)	336 (49.8)	339 (50.2)	10.74	4.03	0.38 (0.23–0.62)	
BRAVO *N* = 881	NR	367 (41.7)	181 (49.3)	186 (50.7)	7.09	8.81	1.24 (0.70–2.19)	0.006
	R	514 (58.3)	268 (52.1)	246 (47.9)	9.12	3.65	0.40 (0.22–0.73)	
CONCERTO *N* = 1456	NR	1015 (69.7)	526 (51.8)	489 (48.2)	6.09	6.56	1.08 (0.74–1.58)	0.033
	R	441 (30.3)	211 (47.8)	230 (52.2)	5.62	2.47	0.44 (0.21–0.94)	

Score group: *NR* non-responders (patients with a response score > − 0.31), *R* responders (patients with a response score ≤ − 0.31)

*EDSS* Expanded Disability Status Scale, *HR* hazard ratio, *CI* confidence interval

*p for treatment by score interaction

**Fig. 4:**
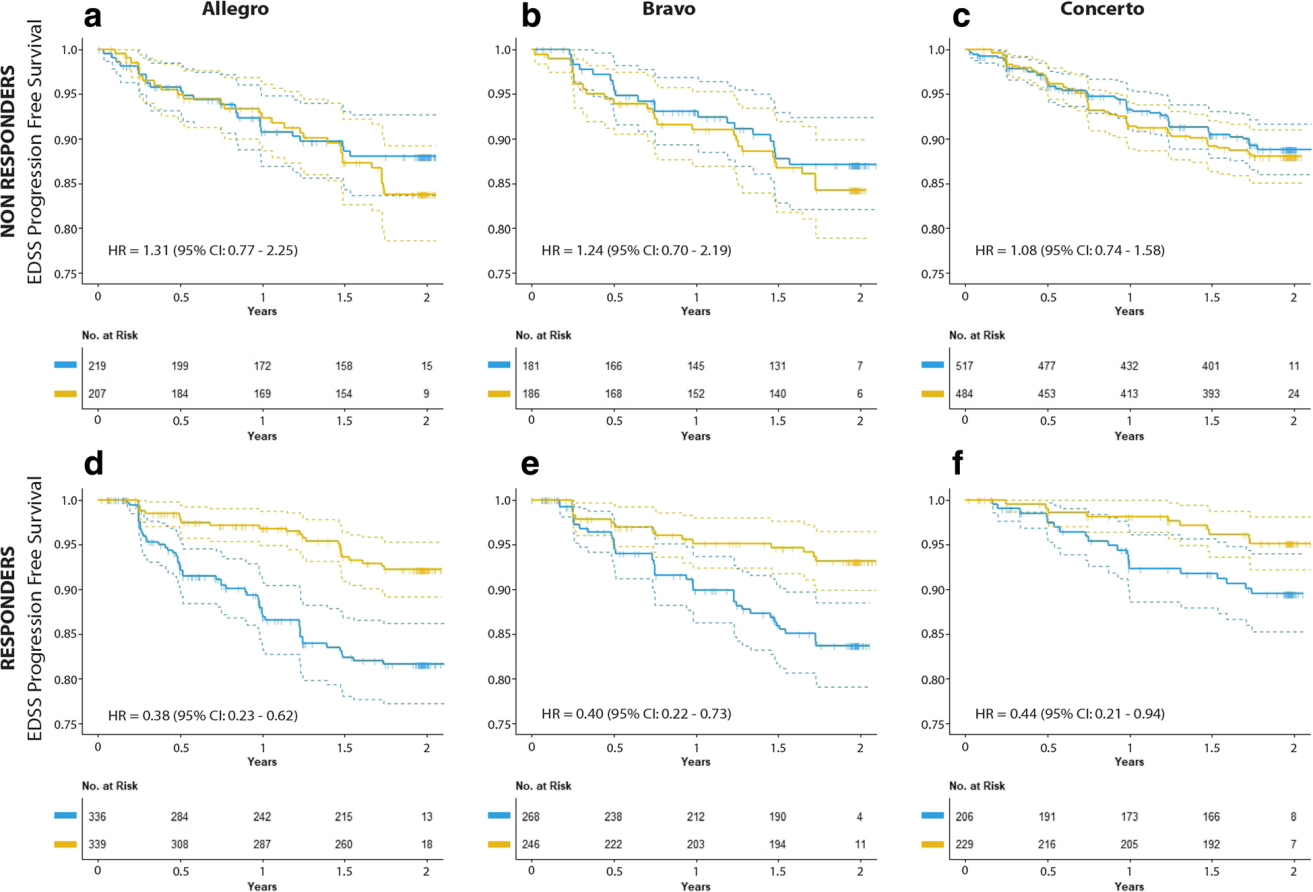
Kaplan–Meier survival curves for the cumulative probability to be free from progression on the Expanded Disability Status Scale (EDSS) confirmed at month 3, in responders (panels **d**-**e**-**f**) and non-responders (panels **a**-**b**-**c**), as defined by the response score, in ALLEGRO, BRAVO, and CONCERTO trials

In the non-responders group, the observed HR for disability progression of laquinimod vs placebo was 1.31 (95% CI 0.77–2.25) in the ALLEGRO trial and HR = 1.24 (95% CI 0.70–2.19) in the BRAVO trial (Fig. 4a, b). In the responders group, the observed HR for disability progression of laquinimod vs placebo was 0.38 (95% CI 0.23–0.62) in the ALLEGRO trial and HR = 0.40 (95% CI 0.22–0.73) in the BRAVO trial respectively (Fig. 4d, e).

### External validation of the response score

These findings were lastly checked against the external validation dataset (CONCERTO study) that was not used in the previous analyses. The response score by treatment interaction was statistically significant (*p* = 0.033) providing indication that also on CONCERTO the response score was able to identify a heterogeneous response to laquinimod. In the non-responders group (69.7% of the patients), the HR for disability progression of laquinimod vs placebo was 1.08 (95% CI = 0.74–1.58), and, in the responders group (30.3% of the patients), the HR for disability progression of laquinimod vs placebo was 0.44 (95% CI = 0.21–0.94) (Fig. 4c, f).

The baseline characteristics of responders and non-responders in the three trials are reported in Table 4.

**Table 4:** Baseline demographic and clinical characteristics of responders and non-responders in the three trials

	ALLEGRO		BRAVO		CONCERTO	
	NR *N* = 426	R *N* = 675	NR *N* = 367	R *N* = 514	NR *N* = 1015	R *N* = 441
Mean age (SD), years	*37.1 (8.5)*	*39.7 (9.4)*	36.6 (8.8)	37.8 (9.8)	*36.0 (8.8)*	*37.3 (9.7)*
Gender, males (%)	*299 (70.2)*	*46 (6.8)*	*260 (70.8)*	*20 (3.9)*	*465 (45.8)*	*0 (0.0)*
Mean number of relapses in the previous year (SD)	*1.60 (0.80)*	*1.03 (0.49)*	*1.55 (0.75)*	*1.13 (0.45)*	*1.44 (0.61)*	*1.04 (0.20)*
Mean disease duration (SD), years	8.22 (6.66)	8.89 (6.87)	6.62 (5.94)	6.92 (6.53)	5.94 (4.23)	5.51 (3.66)
Median EDSS (range)	2.5 (0–5.5)	2.5 (0–6)	*3 (0–5.5)*	*2.5 (0–5.5)*	2.5 (0–5.5)	2.5 (0–5.5)
Number of patients with Gd+ baseline scans (%)	*159 (37.3)*	*316 (46.8)*	123 (33.5)	197 (38.3)	*352 (34.7)*	*226 (51.3)*
Mean T2 lesion volume (SD), log-transformed cm^3^	*1.97 (1.16)*	*1.53 (1.29)*	*1.72 (1.35)*	*1.29 (1.45)*	*1.68 (1.22)*	*1.41 (1.19)*
Mean T1 lesion volume (SD), log-transformed cm^3^	*0.31 (1.80)*	*−0.33 (1.96)*	*0.42 (1.76)*	*− 0.27 (1.90)*	*1.31 (1.31)*	*0.97 (1.29)*
Mean normalized brain volume (SD), cm^3^	*1553.9 (90.5)*	*1599.5 (90.6)*	*1547.5 (91.8)*	*1610 (87.4)*	*1410.4 (91.5)*	*1491.7 (68.1)*

Data reported as mean (standard deviation) and number (percentage) for continuous and categorical variables, respectively (unless otherwise specified). Characteristics presented in italics are those that present a significant difference between respondent and non-respondent (*p* < 0.05)

*NR* non-responders (patients with a response score > − 0.31), *R* responders (patients with a response score ≤ − 0.31), *EDSS* Expanded Disability Status Scale, *GD+* gadolinium-enhancing

## Discussion

When many drugs become available for a disease, new challenges arise for clinicians, based on the possibility to make a choice. On the other hand, the scientific breakthroughs in our understanding of how a person’s unique molecular and genetic profile makes them susceptible to respond to specific treatments for some medical conditions have made it possible to identify patient subgroups who would benefit the most from specific therapies. This is not the case for MS: since the first disease-modifying drug was approved in 1993, the approach to therapies has evolved in the direction of initiating treatment early in the disease course and to switch to a different drug in case of breakthrough disease activity or poor tolerability. Few specific biomarkers (more related to safety rather than efficacy) are available to guide the treatment choice. It is also well recognized that the MS disease course, as well as the drug response of most patients, is highly heterogeneous. Thus, it is not possible to identify drug responders or non-responders in advance, or even retrospectively.

Post hoc subgroup analyses of clinical trials in MS did not find specific markers of response to the approved drugs: the results of a recent meta-analysis [12] including all the post hoc subgroup analyses of clinical trials in RRMS indicated generic predictors of higher response to immunomodulatory treatments, like a younger age, a lower EDSS, and a higher MRI activity measured by the presence of Gd+ lesions on the baseline scan. In this paper, we report a method to create a combination of baseline variables in a continuous score, representing a predicted response to a specific treatment. It is possible, in fact, that the full patient profile made up of the combination of multiple baseline variables is more powerful and more informative than each single characteristic to predict the response to treatments. Interestingly, even if all the three factors mentioned above are included into the response score for this drug in MS, age is included in the opposite direction (larger effect for older patients), when in combination with the other selected factors.

This analysis of the distribution of response to treatment is able to explain the contrasting results of the three trials. The third clinical trial of laquinimod (the CONCERTO trial) was launched to test the disproportionate effect on disability progression detected both in the ALLEGRO (HR = 0.64, *p* = 0.01) and in the BRAVO (HR = 0.69, *p* = 0.06) studies, despite the low activity of the drug on inflammatory markers such as relapses and MRI endpoints. The present analysis highlighted that there is a subgroup of patients (that can be identified by the set of baseline variables combined in the response score) with a larger response to laquinimod (about 60% of patients both in ALLEGRO and in BRAVO) leading to an EDSS progression risk reduction higher than 60%, while there is a subgroup with no effect (about 40% of patients). The proportion of non-responding patients is higher in the CONCERTO study (around 70%) while those with a favorable response score are just the 30%, due to differences in baseline characteristics as compared to those of the cohort enrolled in the previous studies. In the subgroup of responding patients, the EDSS progression risk reduction is of the same magnitude as in the two previous trials. Therefore, this analysis suggests that the CONCERTO trial failed to demonstrate a treatment benefit mainly because the proportion of non-responding patients enrolled in this study was higher.

It is important to note that the model selection was based here just on objective model performance criteria. Other criteria, more focused on biological/medical knowledge or driven by parsimony and simplicity of implementation in clinical practice, could be considered in the final step for model selection.

## Conclusions

This study demonstrates the possibility to refine and personalize the treatment effect estimated in randomized clinical trials by using the baseline demographic and clinical characteristics of the included patients. This is an easy and affordable method toward therapy personalization, suggesting patient profiles related to a larger benefit from a specific drug.

This analysis was conducted on trials of a drug that was not approved on the market; therefore, the implications of this study are not for the specific treatment: the main aim of this analysis is to show the potential of the method and to stimulate similar exercises (with practical implications) on approved therapies. This analysis could be run, in fact, on clinical trials testing every drug approved for a specific condition to create a set of drug-specific scores. This effort calls for pharmaceutical companies to allow the re-analysis of clinical trial data and the publication of results that might indicate patient characteristics of drug responders. Ultimately, the value of personalized treatment approaches should be confirmed by evidence generated by prospective clinical trials which incorporate the response scores.

## Data Availability

The data that support the findings of this study are available from Teva Pharmaceutical Industries, but restrictions apply to the availability of these data, which were used under license for the current study, and so are not publicly available. Data are however available from the authors upon reasonable request and with permission of Teva Pharmaceutical Industries.

## Abbreviations

95% CI: 95% confidence interval
CHMP: Committee for Medicinal Products for Human Use
EDSS: Expanded Disability Status Scale
Gd+: Gadolinium-enhancing
HR: Hazard ratio
MS: Multiple sclerosis
NBV: Normalized brain volume
NR: Non-responders
R: Responders
RCT: Randomized clinical trial
RR: Relapsing–remitting
SD: Standard deviation
